# Composition and structure of the culturable gut bacterial communities in *Anopheles albimanus* from Colombia

**DOI:** 10.1371/journal.pone.0225833

**Published:** 2019-12-02

**Authors:** Yadira Galeano-Castañeda, Paula Urrea-Aguirre, Stefani Piedrahita, Priscila Bascuñán, Margarita M. Correa

**Affiliations:** Grupo de Microbiología Molecular, Escuela de Microbiología, Universidad de Antioquia, Medellín, Colombia; Instituto Nacional de Salud Pública, MEXICO

## Abstract

The understanding of factors affecting the gut bacterial communities in malaria vectors is essential for the design of vector control interventions, such as those based on a paratransgenic approach. One of the requirements of this method is the availability of bacteria from the mosquito susceptible to culture. Thus, the aim of this study was to evaluate the composition and structure of the culturable gut bacterial communities in field mosquitoes *Anopheles albimanus* from Colombia, in addition to generate a bacterial collection to further analyze microbial functional activity. Gut bacteria were isolated from *An*. *albimanus* larvae and adult mosquitoes collected in localities of the Atlantic and Pacific Coasts. The bacterial isolates were grouped in 28 morphospecies that corresponded to three phyla, three classes, nine families and 14 genera. The larvae guts from San Antero (Atlantic Coast) and Buenaventura (Pacific Coast) shared the genera *Bacillus* and *Lysinibacillus* and in adults, *Bacillus* and *Bacillus cereus* Group were registered in both localities. Gut bacterial richness was higher in adults from the Pacific with respect to the Atlantic Coast, while larval richness was similar in samples of both coasts. The Shannon index indicated uniformity in morphospecies abundances in both localities. Finally, the characterization of morphospecies from the gut of *Anopheles albimanus* mosquitoes from Colombia by culture-dependent methods complemented with 16S rRNA gene sequencing allowed the definition, at a finer resolution, of the composition and structure of these microbial communities. In addition, the obtained bacterial culture collection will allow further evaluation of the microorganisms for their potential as biocontrol agents.

## Introduction

The mosquito gut microbiota highly influences the development and biological functions of insects [1]. These bacteria confer an extended phenotype that contributes to vital functions such as nutrient assimilation [2], peritrophic membrane formation [3] [4] and modulation of vector capacity [5]. Particularly in *Anopheles* mosquitoes, some gut bacteria interfere with *Plasmodium* parasite development [6], through mechanisms such as enzyme and toxin production [7] [8] [9] or by direct bacterial interaction with the mosquito immune response [10]. With the possibility of bacteria inhibiting the *Plasmodium* parasite, a strategy was proposed in which bacteria recovered from mosquitoes are genetically modified to confer properties that prevent the vector from transmitting the pathogen; these paratransgenic bacteria are then introduced into vector populations in the wild [11].

Characterization of *Anopheles* gut microbiota for the elucidation of bacterial functions and antiparasitic activity requires the isolation by culture of the gut native bacteria. To date, various bacterial candidates with paratransgenic potential have been described, such as a *Serratia* isolate that successfully colonizes the mosquito gut [7], female ovaries, and male accessory glands [12], it also has the capacity to inhibit *Plasmodium* development [7]. Similarly, the *Enterobacter* strain Esp_Z increases *Anopheles* resistance to *Plasmodium* infection by the production of reactive oxygen species (ROS) [9]. Furthermore, an *Enterobacter cloacae* strain stimulates the expression of mosquito serpins, specifically SRPN6, which potentiates *Anopheles stephensi* immune response against *Plasmodium falciparum* [10]. These findings have led to a novel approach based on paratransgenesis in which bacteria isolated from mosquitoes are potentiated to generate antiparasitic activity [13]. Until now, the evaluation of candidate bacteria with potential for a paratransgenic control has been performed under laboratory conditions [9] [10]. The most studied model for such approach is the tripartite *Asaia*, *Anopheles* and *Plasmodium*. The genus *Asaia* has been detected in larvae and adults of *Anopheles gambiae* and *An*. *stephensi* guts [14] [15] [16]. Modified *Asaia* strains (GFP-*Asaia* and DsRed-*Asaia*) were able to colonize the midgut, salivary glands and reproductive organs in male and female mosquitoes [14] [15], and vertical transmission was evidenced [15] [17]. In addition, *Asaia* efficiently increased the mosquito immune response and co-localized with *Plasmodium-*parasites in the midgut and salivary glands, leading to a decrease in parasite abundance [18]. Even the gained knowledge in paratransgenic bacterial candidates, the challenge consists in translating the results to field conditions. In the case of *Asaia*, studies in semi-natural settings demonstrated this bacterium stability in *An*. *stephensi* over time, horizontal and vertical transmission capacity, characteristics that would facilitate its dissemination in the mosquito population [19].

Among the features that a bacterial candidate should fulfill for the success of a paratransgenic approach are, 1. Ability to colonize and persist in the mosquito gut environment where it competes with the native gut bacterial communities [7], 2. Survival during mosquito trans-stadial passage, and 3. Capacity to grow in culture [20]. Therefore, the availability of bacterial cultures will ensure counting with the microorganism to perform additional analyses. Even though, various paratransgenic bacterial candidates have been described, it is relevant to consider that bacterial communities in the mosquito gut are dynamic, implying that their structure and composition are determined by factors such as geography [21] [22] and mosquito stage [23] [24]. Most studies regarding the mosquito gut microbiota have been conducted in the Asian and African malaria vectors [25] [7] [10] and very few have evaluated the gut bacterial communities of the Latin American vectors [22] [26] [27]. Furthermore, the structure and composition of the culturable bacterial communities have not been explored in these vectors. The use of these bacteria in a paratransgenic control strategy will require knowledge on the native bacterial communities of the malaria vectors and a throughout understanding of how they are influenced by factors such as geographical origin and developmental stage. Therefore, the aim of this study was to evaluate the composition and structure of the culturable gut bacterial communities of the Colombian main malaria vector *Anopheles albimanus*, according to geographical origin and mosquito stage. This information and the availability of a bacterial culture collection will allow testing microbial candidates for their functionality in the search for autochthonous paratransgenic candidates.

## Material and methods

### Collection sites

**Fig 1:**
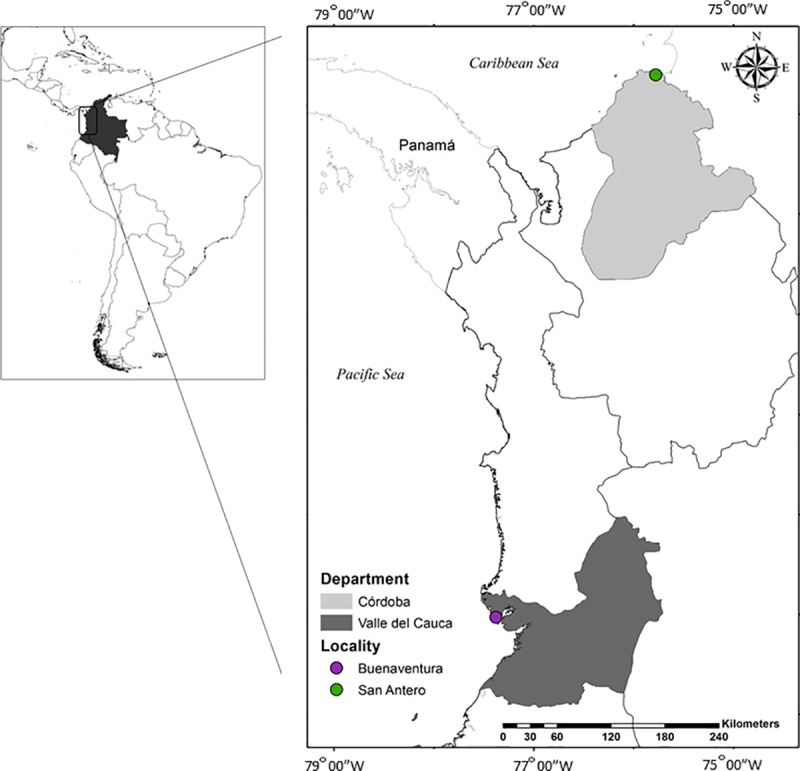
Collection sites for *An*. *albimanus* larvae and adults. Collections were performed in localities of the municipalities: San Antero in Córdoba Department, Atlantic Coast (09°23'36.8''N; 75° 46'11,8''W) and Buenaventura in Valle del Cauca Department, Pacific Coast (3°57′59.9″N; 77°22′42.7″W).

*Anopheles* mosquitoes were collected during the years 2014 and 2015 in localities of the Colombian municipalities San Antero (09° 23'36.8''N;75°46'11.8''W) in the Atlantic Coast and Buenaventura (03° 57'59.9''N; 77° 22'42 ', 7''W) in the Pacific Coast (Fig 1). The sites were selected considering the distribution of *An*. *albimanus* in Colombia, which is mainly in these coasts and they encompass regions presenting different ecological features. The Pacific Coast is characterized by tropical forest, alluvial valleys and flooded plains and lakes, it has one of the highest precipitation levels in the world (> 5,000 mm annually). During specimen collection in Buenaventura, humidity records ranged between 89% and 94%, with an average temperature of 27°C. The Atlantic Coast is characterized by contrasting topography, climate and vegetation, with deserted regions, savannas and important river valleys; it presents high temperatures and evident rainfall and dry seasons [28]. Relative humidity in San Antero ranged between 75% and 80%, and the average temperature was 28°C. Collections were performed for three nights, between 18:00–00:00 h, using human landing catches under a protocol and informed consent agreement reviewed and approved by a University of Antioquia Institutional Review Board (Comité de Bioética Sede Investigación Universitaria, approval document 15-41-580). The collection sites in both sampled localities were rural.

### Identification and processing of *Anopheles albimanus* specimens

Specimens were identified as *An*. *albimanus* based on wing and hind leg characters, following a taxonomic key [29]. In addition, *Anopheles* larvae were collected using a dipper and fourth stage larvae were stored in DNA preservation solution (Zymo research). For each locality, in the last sampling day, 11 mosquitoes and 11 larvae were taken for bacterial isolation, identification and diversity analyses. Mosquito guts were dissected in the field under sterile conditions [20] [24], after rinsing with 90% ethanol for 3 minutes and washing twice with 0.9% sterile saline solution. The dissected guts were preserved in sterile saline solution, at 4°C, until processing in the laboratory. The last rinse was used as the control for mosquito surface sterility, but no growth was obtained; therefore, contamination during the dissection process was ruled out. Mosquito remnants were used for genomic DNA extraction [30], and *Anopheles* species confirmation by a Polymerase Chain Reaction—Restriction Fragment Length Polymorphism of the Internal Transcribed Spacer 2—PCR-RFLP-ITS2 [31].

### Processing of dissected guts, bacterial isolation and characterization by phenotypic, biochemical and antibiotic resistance tests

Larval and adult guts were macerated individually in 50 μL of 0.9% sterile saline solution. For characterization of the bacterial communities by culture-dependent methods, one μL samples were used to inoculate blood agar and nutritive agar plates, in duplicates, which were incubated at room temperature for 24–48 h. Based on the characteristics of the colonies, re-isolations were carried out in the same type of media until axenic cultures were obtained. Additionally, 45 μL were used for DNA extraction and DNA pools from five mosquito guts were used to detect *Plasmodium* natural infection by a nested PCR [32].

The colonies from axenic cultures were described based on characteristics such as shape, edge, size, elevation, surface, color and brightness; also, Gram staining was performed. These results guided the phenotypic tests. Control strains were *Staphylococcus aureus*, *Bacillus cereus*, *Escherichia coli* and *Pseudomonas aeruginosa* (kindly provided by Laboratorio. MIA-UdeA). Gram-positive bacilli were tested for spore formation, mobility by the sulfide indole motility medium (SIM) and hemolysis in blood agar. For Gram-negative bacilli, lactose and sucrose fermentation, gas production in triple sugar iron agar (TSI) and cytochrome oxidase activity were tested using BD BBL^™^ p-aminodimethylaniline disks (Becton, Dickinson and Company); as well as hydrogen sulfide production and motility using SIM agar. Tests for Gram-positive cocci included hemolysis type in blood agar, catalase and coagulase activities and mannitol fermentation. And, for Gram-negative cocci and coccobacilli, cytochrome oxidase, catalase and sugar fermentation were performed.

In order to improve the physiological characterization of isolates, a screening for antibiotic resistance was performed. The determination of the antibiotic resistance profile is important for future *in vivo* mosquito-colonization tests. Axenic colonies were inoculated in nutritive agar supplemented with the antibiotics, 15 μg/μL tetracycline, 100 μg/μL ampicillin and 20 μg/μL erythromycin [33].

### DNA extraction from bacterial isolates, 16S rRNA gene amplification and sequencing

A sample of each colony was inoculated in 5 mL Luria Bertani enrichment medium and incubated for 18 to 24 h at room temperature with shaking. After growth, an aliquot was stored in glycerol at a 10% final concentration, at -80°C. The remaining culture was transferred to a 1.5 mL vial, centrifuged at 13,000 rpm for 5 min, the supernatant discarded and the pellet processed for DNA extraction using the DNAeasy Blood & Tissue kit (Qiagen), following manufacturer recommendations. The lysis buffer for Gram positive bacteria contained lysozyme (20 mg/mL) and 25 μl of Proteinase K.

The 16S rRNA gene was amplified using universal primers 27F and 1492R [34]. The 25 uL reaction mixture contained: DNA 20 ng/ μl, 0.05 μM of each primer, 1 unit of Taq DNA polymerase (*Taq* polymerase Bioline), 0.4 mM of dNTPs, 1.5 mM MgCl_2_, 1X PCR buffer and Milli-Q water. PCR conditions consisted of an initial denaturation step of 94°C for 3 min; 30 cycles of each of the following steps, denaturation at 94°C for 30 s, alignment at 53°C for 1 min, elongation at 72°C for 30 s and a final extension at 72°C, for 10 min. Amplified products were sequenced by the Sanger method with universal primer 800R: 5′- TAC CAG GGT ATC TAA TCC-3'. Subsequently, a basic local alignment search tool (BLAST) was performed with the MUSCLE algorithm incorporated in Geneious Version 8.0.3 [35] against the sequences reported in Genbank.

### 16S rRNA sequence analysis

The sequences were edited in the Geneious 8.0.3 software [35]. To assign the taxonomic identity of each sequence, a BLAST was performed with the sequences reported in GenBank and the NCBI using the MUSCLE algorithm incorporated into the program. To determine bacterial taxonomic status, a Bayesian analysis was carried out with the V2 to V4 regions of the 16S rRNA gene, performing partitions of the regions. To generate a cluster tree diagram that represented bacterial relationships, four Monte Carlo Markov chains were run for five million generations, sampling every 200 generations, discarding 25% of the runs as burn-in. The *Ureaplasma parvum* 16S rRNA gene was used as the outgroup. These analyses were executed in the Mr. Bayes 3.2 program [36]. The best DNA substitution model for each data set was selected based on the Akaike Information Criterion (AIC) in jModeltest 2 [37].

### Diversity analyses

Bacterial communities diversity was evaluated using the Simpson diversity indexes (1-D), Shannon-Weaver (H'), Equitability (J) and Dominance index (D) using the PAST software v. 2.07 [38]. To estimate sampling effectiveness, species accumulation curves were constructed in EstimateS v. 9.1.0 [39], using presence/absence estimators such as Chao 2, incidence-based coverage (ICE), Jack 1 and Jack 2, S (est), Bootstrap, and unique and duplicate assessments. In addition, similarities between the *An*. *albimanus* gut bacterial communities from the San Antero and Buenaventura collection sites (locality comparison), and between stages, were assessed by the Jaccard estimator with 1000 permutations, using the UPGMA algorithm in PAST v. 2.07.

## Results

### Composition and structure of *An*. *albimanus* gut bacterial communities

The composition of *An*. *albimanus* gut bacterial communities was assessed by taxonomic assignment based on 16S rRNA gene sequences. From the 11 mosquitoes and 11 larvae processed by locality, a total of 40 bacterial morphotypes were obtained, 25 from larval guts and 15 from the mosquito guts. In an attempt to determine the influence of the gut bacteria in parasite presence, mosquito natural infection with *P*. *vivax* or *P*. *falciparum* was determined; however, no further analysis could be performed since the mosquitoes were not found infected.

**Fig 2:**
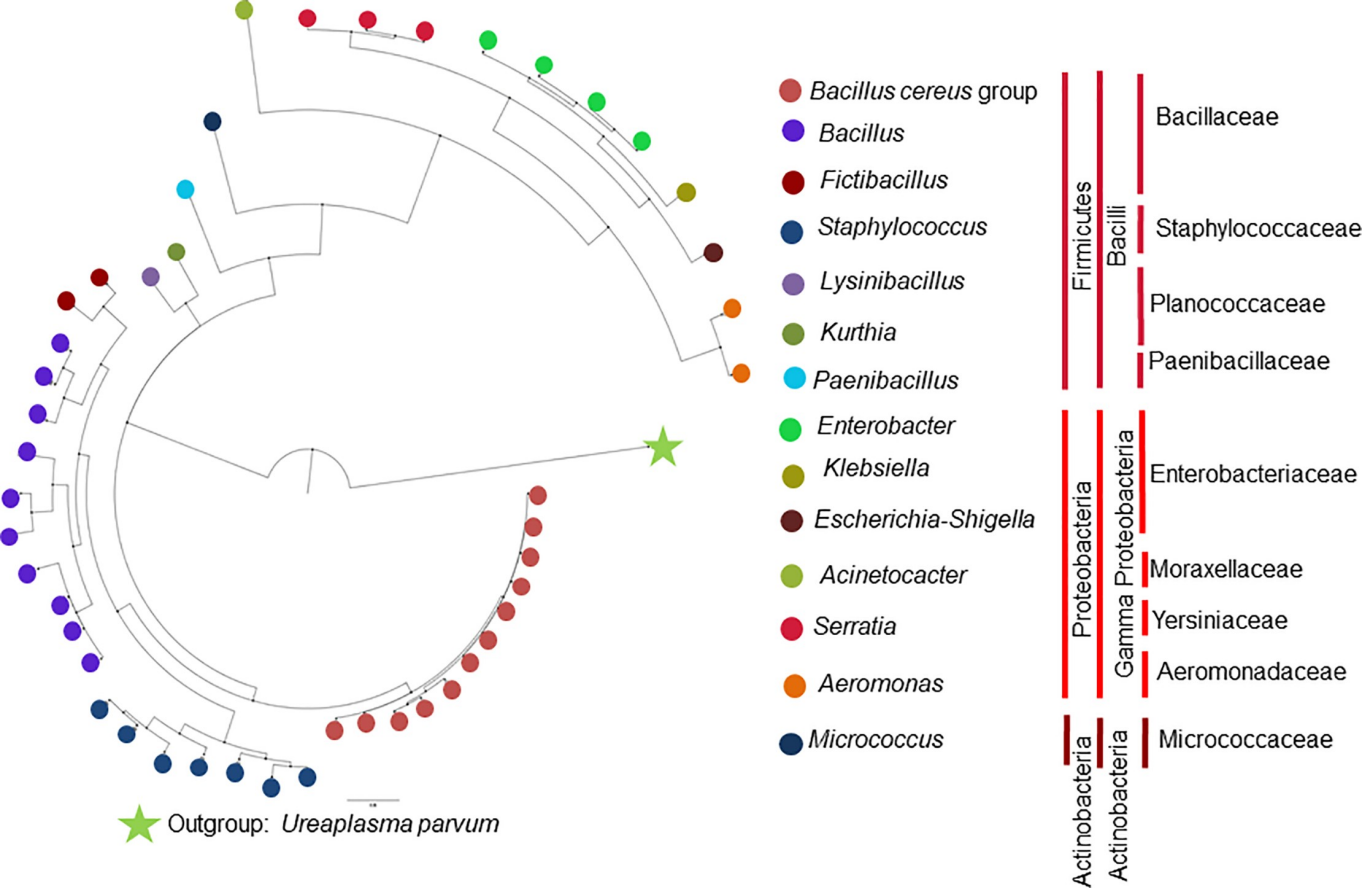
Dendrogram based on Bayesian inference. The cluster tree diagram that represents bacterial relationships was based in 16S rRNA gene sequences. *Ureaplasma parvum* was used as the outgroup. Colored circles represent members of bacterial genera. Paralel vertical lines from left to right define phylum, class and family. Branch support ranged between 0.93 and 1.

A Bayesian analysis based on 16S rRNA grouped the morphotypes into three phyla, three classes, nine families and 14 genera (Table 1, Fig 2). Regarding gut bacterial distribution according to locality, it was found that the phyla Firmicutes and Proteobacteria were present in both localities, San Antero and Buenaventura and Actinobacteria was only detected in San Antero. At the family level Bacillaceae, Planococcaceae and Enterobacteriaceae were found in both localities, while Staphylococcaceae, Moraxellaceae, Yersiniaceae and Paenibacillaceae were detected in Buenaventura, but not in San Antero, while Aeromonadaceae and Micrococcaceae were only observed in mosquitoes from San Antero (S1 Table).

**Table 1:** Taxonomic classification of bacterial gut isolates from *Anopheles albimanus* larvae and adults based on 16S rRNA gene sequences.

Phylum	Class	Family	Genus
Firmicutes	Bacilli	Bacillaceae	*Bacillus cereus* Group
			*Bacillus* spp.
			*Fictibacillus* spp.
		Staphylococcaceae	*Staphylococcus* spp.
		Planococcaceae	*Kurthia* spp.
			*Lysinibacillus* spp.
		Paenibacillaceae	*Paenibacillus* spp.
Proteobacteria	Gamma Proteobacteria	Enterobacteriaceae	*Escherichia-Shigella*
			*Klebsiella* spp.
			*Enterobacter* spp.
		Yersiniaceae	*Serratia* spp.
		Moraxellaceae	*Acinetobacter* spp.
		Aeromonadaceae	*Aeromonas* spp.
Actinobacteria	Actinobacteria	Micrococcaceae	*Micrococcus* spp.

**Fig 3:**
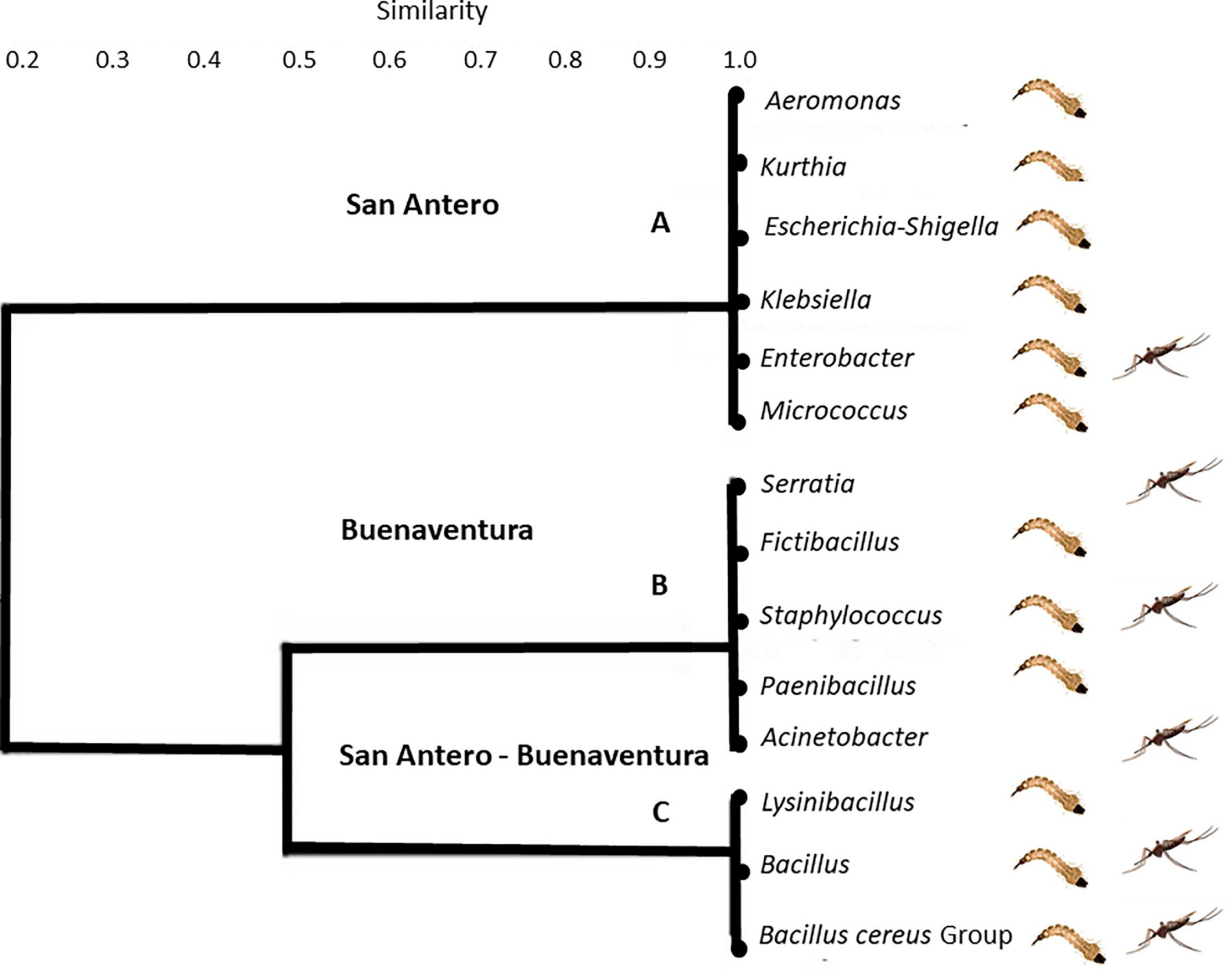
Dendrogram based on Jaccard distances using the UPGMA algorithm with 1000 replicas. Bacterial genera distribution according to locality and stage. The genera detected in the localities sampled in San Antero (Atlantic Coast) and Buenaventura (Pacific Coast) and in both. Larva and mosquito figures indicate the mosquito stage in which bacterial genera were detected.

Furthermore, the 40 bacterial morphotypes were classified into 14 genera that were differentially distributed according to locality and mosquito stage (Fig 3). In San Antero, the genera *Bacillus* and *Enterobacter* were detected in both, larvae and adults; *Kurthia*, *Lysinibacillus*, *Escherichia-Shigella*, *Klebsiella*, *Aeromonas* and *Micrococcus* were only found in larvae, while the *Bacillus cereus* Group was only recorded in adults (S1 Table, S1 Fig). In Buenaventura, the genera *Bacillus*, *Bacillus cereus* Group, *Staphylococcus* and *Lysinibacillus* were detected in both, larvae and adults. *Serratia* and *Acinetobacter* were only detected in adults, whereas *Paenibacillus* and *Fictibacillus* were only observed in the larvae (S1 Table, S1 Fig).

The *An*. *albimanus* larval guts from San Antero and Buenaventura shared bacteria of the genera *Bacillus* and *Lysinibacillus*, while, *Escherichia-Shigella*, *Klebsiella*, *Aeromonas*, *Micrococcus* and *Enterobacter* were unique to San Antero, and *Fictibacillus*, *Paenibacillus*, *Bacillus cereus* Group and *Staphylococcus* to Buenaventura (S1 Table, S2 Fig). In adults, the genera *Bacillus* and *Bacillus cereus* Group were registered in both localities; while, *Enterobacter* only in San Antero, and *Acinetobacter*, *Staphylococcus*, *Lysinibacillus* and *Serratia* in Buenaventura (S1 Table, S2 Fig).

### Structure of the gut bacterial communities based on morphospecies

**Fig 4:**
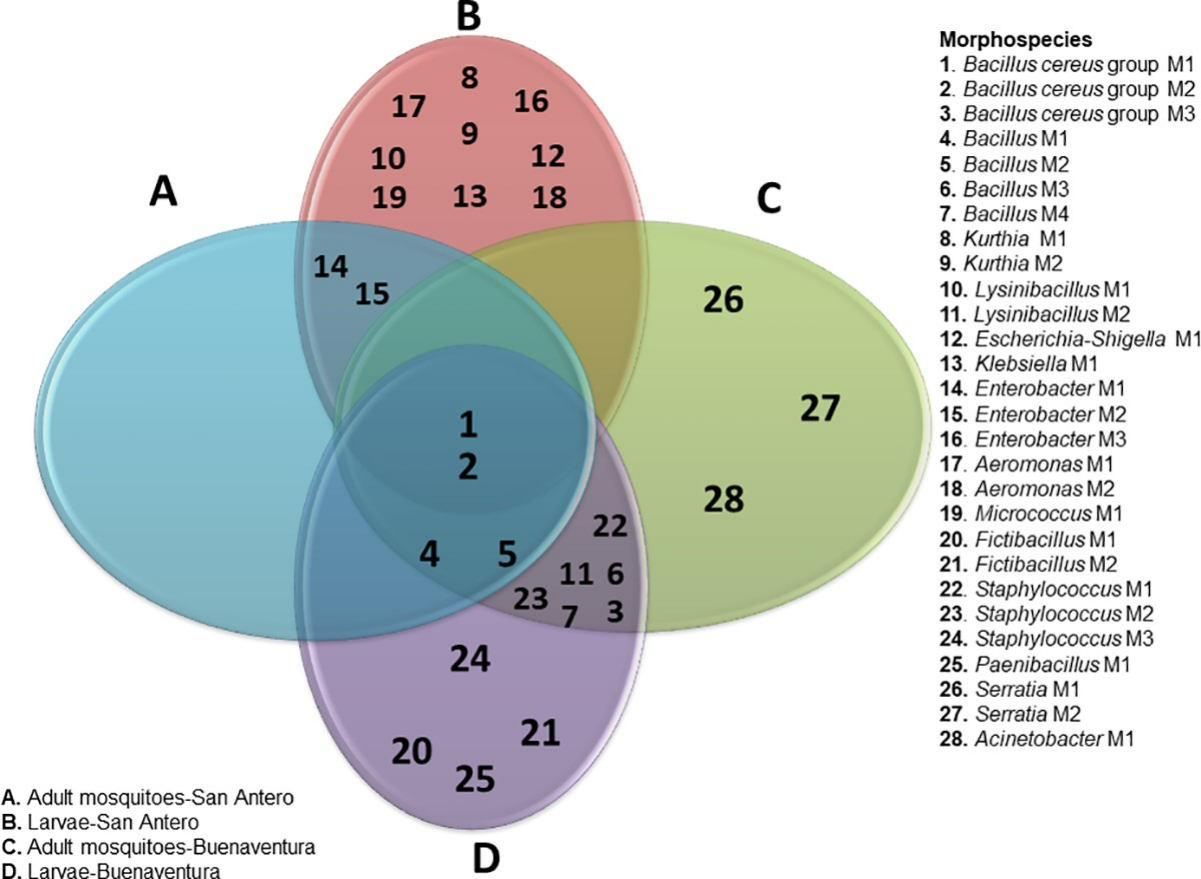
Venn diagram showing shared morphospecies among larvae, adults and localities, San Antero and Buenaventura. The diagram shows the 28 morphospecies detected by locality and mosquito stage. In the intersections are the morphospecies shared by groups, locality and stage (represented by numbers).

**Fig 5:**
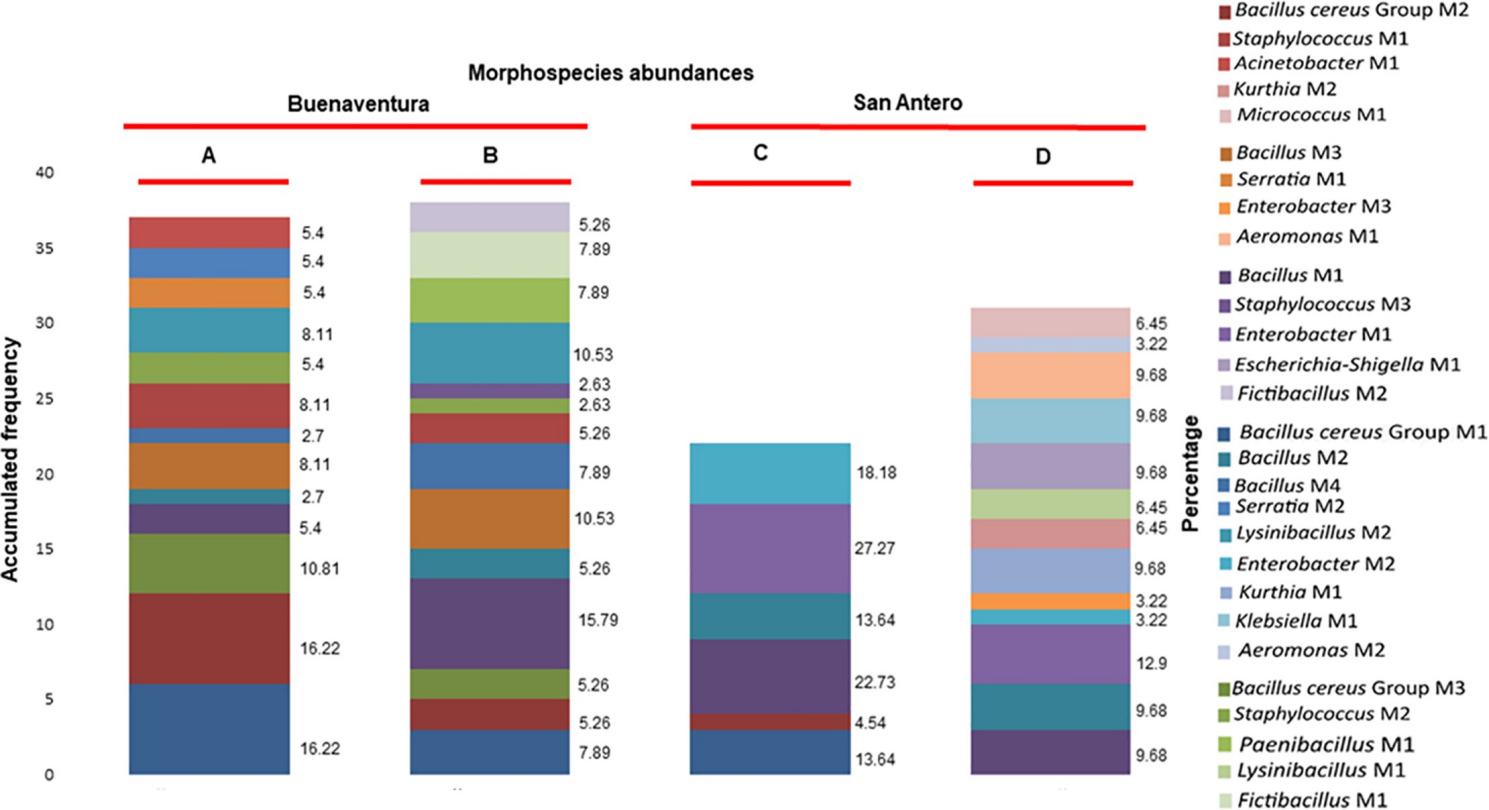
Morphospecies abundances in gut bacterial communities from larvae and adult *Anopheles albimanus* from San Antero and Buenaventura. Morphospecies abundances are given in percentages, the values are shown at the right side of the bars; the accumulated frequency is also shown (left, Y axis). Colors represent morphospecies. (A) larvae form Buenaventura (Pacific Coast), (B) adults form Buenaventura, (C) larvae from San Antero (Atlantic Coast), (D) adults of San Antero.

A concatenated analysis of the taxonomic assignment based on 16S rRNA gene sequences and phenotypic data, helped to classify the 40 morphotypes in 28 morphospecies (M). *Bacillus cereus* Group-M1 and M2 were the only morphospecies shared in larvae and adults of San Antero and Buenaventura (Fig 4). The most abundant morphospecies were, *Bacillus cereus* Group-M1 in Buenaventura and *Enterobacter*-M1 in San Antero (Fig 5). In Buenaventura, the most abundant morphospecies in adults were the *Bacillus cereus* Group- M1 and *Bacillus-*M2, each representing 16.22% of the communities (Fig 5A). The most abundant morphospecies in larvae was *Bacillus*-M1 (15.79%), followed by *Lysinibacillus*-M2 and *Bacillus*-M3 (10.53% each) (Fig 5B). Interestingly, in larvae and adult mosquitoes of San Antero, the predominating morphospecies was *Enterobacter*-M1 with 27.27% in adults and 12.9% in larvae (Fig 5). In adults, the second most abundant morphospecies was *Bacillus*-M1 with 22.73% (Fig 5C), while in larvae, *Escherichia-Shigella*-M1, *Klebsiella*-M1, *Aeromonas*-M1, *Bacillus*-M1, *Bacillus*-M2 and *Kurthia*-M1 followed in abundance with 9.68% each (Fig 5D).

**Fig 6:**
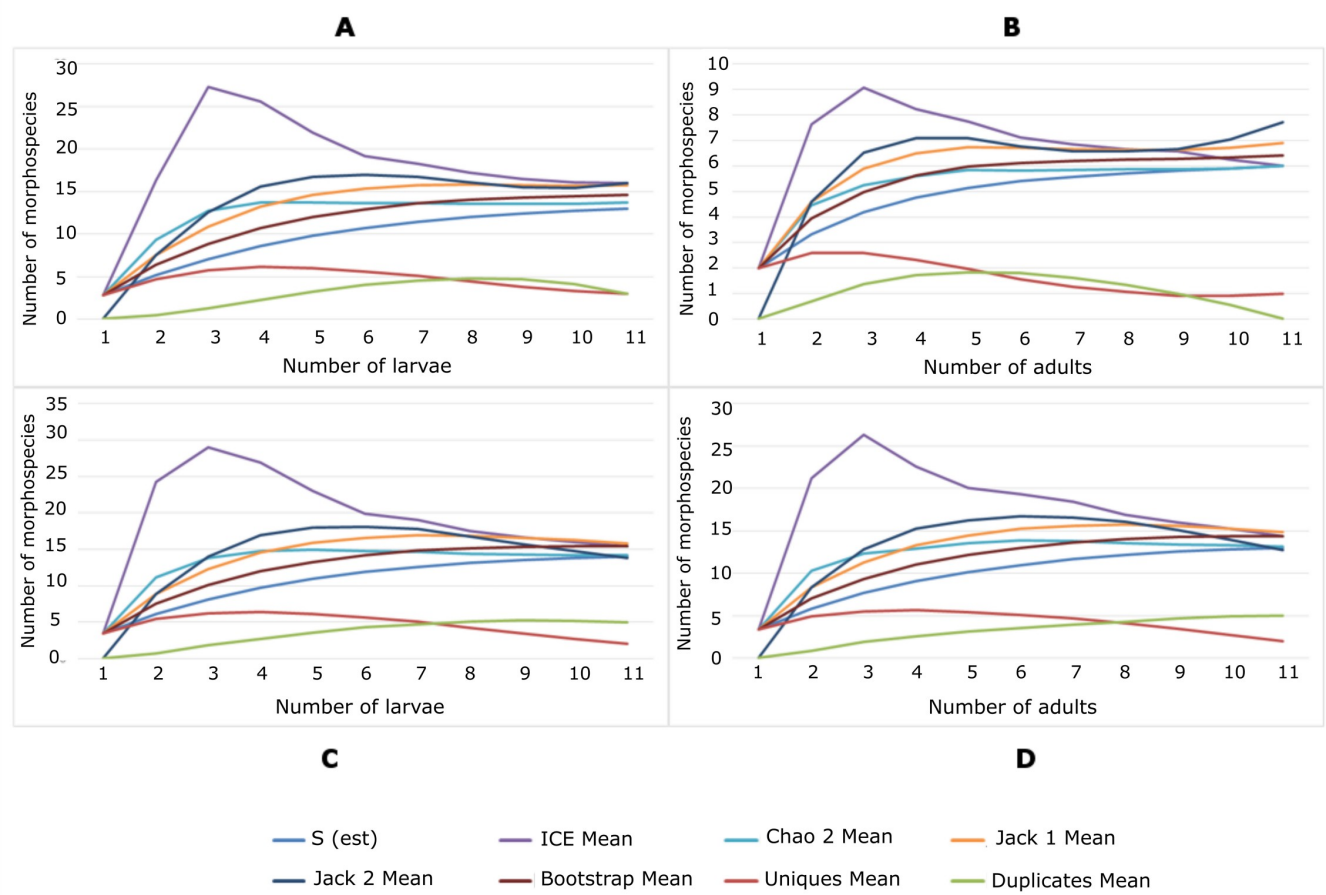
Morphospecies accumulation curves. The X axis represents the number of samples, larvae or adults and the Y axis the number of morphospecies detected. Colored lines corresponds to the indexes evaluated by locality and stage. (A) San Antero (Atlantic Coast)—larvae, (B) San Antero—adults, (C) Buenaventura (Pacific Coast)—larvae, (D) Buenaventura—adults.

Species accumulation curves reached the asymptote, which indicated that the sampling effort, for locality and mosquito stage, was enough to perform the subsequent structure analyses of the gut bacterial communities (Fig 6A–6D). At the locality level, the structure of the adult gut bacterial communities had a greater richness in Buenaventura (S = 13) compared to San Antero (S = 6) and there were no dominant morphospecies. Furthermore, the Shannon index indicated uniformity in morphospecies abundance distribution. The Equitability and Simpson diversity indexes showed that the communities were diverse (Table 2). At the locality and stage levels, in San Antero, a greater richness (S) was found among the bacterial communities of the larvae with respect to the adults; however, the abundances in both stages were homogeneous. In Buenaventura, similar richness values were found for both mosquito stages with no dominance (D) and a homogeneous equity (J) (Table 2).

**Table 2:** Alpha diversity indices for *An*. *albimanus* gut bacterial communities.

Index	San Antero		Buenaventura	
	Larval guts	Adult guts	Larval guts	Adult guts
**Specific richness (S)**	13	6	14	13
**Records number**	31	22	32	37
**Dominance (D)**	0.08845	0.1983	0.08726	0.1001
**Shannon-Weaver (H')**	2.483	1.685	2.533	2.425
**Simpson (1-D)**	0.9116	0.8017	0.9127	0.8999
**Equitability (J)**	0.9681	0.9404	0.96	0.9456

Note: Higher gut bacterial richness was detected in larvae than in adults. No dominant morphospecies was detected in the gut bacterial communities from larvae and adults of San Antero (Atlantic Coast) and Buenaventura (Pacific Coast).

### Antibiotic resistance determination

**Fig 7:**
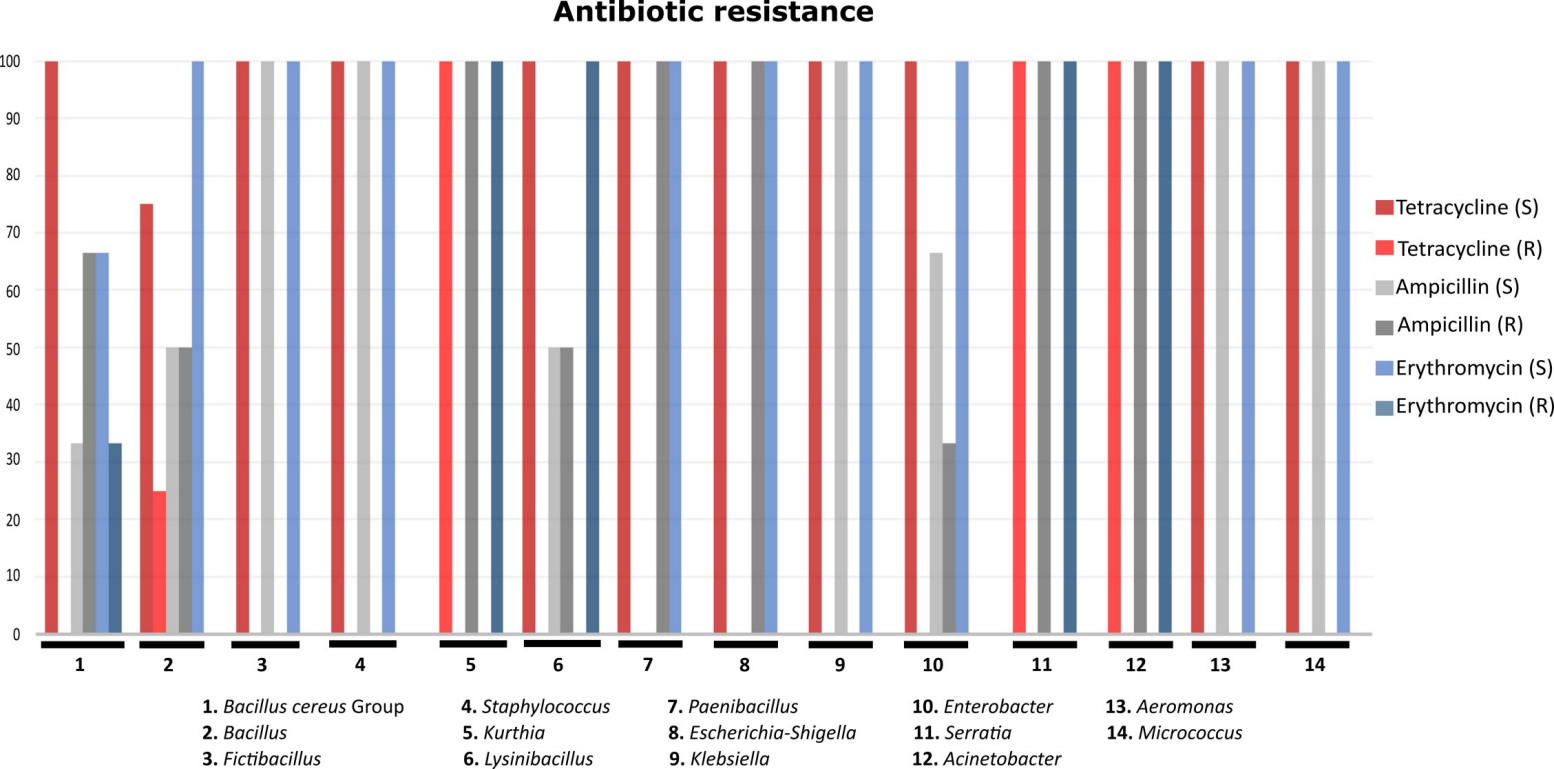
Antibiotic resistance chart. The X axis shows the morphotypes of each genus; the Y axis, the percentage of antibiotic resistant and sensitive morphotypes. (S) Sensitivity, (R) resistance.

Determination of antibiotic resistance showed that 25% (*n*: 1/4) of the morphotypes within the *Bacillus* genus and all of the *Kurthia* (*n*: 2/2), *Serratia* (*n*: 2/2), and *Acinetobacter* (*n*: 1/1) genera were tetracycline resistant. While, all *Paenibacillus* (*n*: 1/1) and *Escherichia-Shigella* (*n*: 1/1), and 33% of the *Enterobacter* (*n*: 1/3) morphotypes were ampicillin resistant. Finally, 33% of the *Bacillus cereus* Group (*n*: 1/3) and 100% of the *Lysinibacillus* morphotypes (*n*: 2/2) were resistant to erythromycin. Of notice, 100% of the morphotypes belonging to the *Kurthia* (*n*: 2/2), *Serratia* (*n*: 2/2), and *Acinetobacter* (*n*: 1/1) genera showed resistance to all the antibiotics evaluated (Fig 7).

## Discussion

In this study, the composition and structure of culturable gut bacterial communities of the Colombian main malaria vector *An*. *albimanus* were evaluated. In general, higher bacterial richness was detected in the larva with respect to the adult stage; also, community composition and structure differed according to geographical origin. Furthermore, this is the first study on the *An*. *albimanus* gut bacterial microbiota that reports the genera *Acinetobacter*, *Lysinibacillus*, *Staphylococcus*, *Aeromonas*, *Klebsiella*, *Paenibacillus*, *Fictibacillus*, *Bacillus*, members of the *Bacillus cereus* Group, *Kurthia*, *Micrococcus* and *Escherichia-Shigella*. Regarding bacterial community structure, the fact that the species accumulation curves reached the asymptote indicated that the sampling effort for locality and mosquito stage was enough to perform the subsequent structure analyses of the gut bacterial communities using diversity indexes. It is known that high throughput sequencing permits detection of a high bacterial richness; however, results showed that culture dependent methods allow detecting, and also isolating, an adequate number of morphospecies to understand the effect of factors such as developmental stage and locality, on *An*. *albimanus* gut microbiota.

With respect to bacterial abundance, the Firmicutes phylum was the most abundant. This result differs from reports for other medically important mosquitoes such as *Culex quinquefasciatus*, *Aedes* spp. and *Anopheles* spp. in which Proteobacteria was the dominant phylum among their gut bacterial communities [24] [40] [41]. But similar to the present results, in *Anopheles* spp. from Vietnam, the genera of Firmicutes phylum *Staphylococcus* and *Bacillus* predominated; and in common with the presents study, mosquitoes were collected in the rainy seasons [42]. Several Proteobacteria genera have been reported with potential for paratransgenic control, eg. *Enterobacter* and *Serratia* [7] [10] [27]. These two genera were also detected among the *An*. *albimanus* gut bacteria and have been described in various Diptera serving important biological functions. As such, *Enterobacter*, *Acinetobacter* and *Serratia* participate in blood digestion through hemolysin production [43]. In this study, one *Enterobacte*r and all *Serratia* morphotypes presented alpha hemolysis in blood agar, which suggests their possible role in hemoglobin digestion in the *An*. *albimanus* gut. Of notice, of the genera of the Firmicutes phylum predominating among the *An*. *albimanus* gut bacteria, *Bacillus* and *Lysinibacillus* have been described with larvicidal activity [44] [45]. Moreover, the *Fictibacillus* detected in *An*. *albimanus* larval guts constitutes the first report of this genus in *Anopheles*. The fact that *Fictibacillus* has been isolated from river waters [46] and marine sediments [47], suggests its acquisition from the water of the larval habitat during feeding. In addition, it is known that *An*. *albimanus* larvae tolerate high salinity waters [48] and during mosquito collection it was observed that *An*. *albimanus* positive larval habitats were located close to the sea and occasionally, seawater mixed with water from the habitat. Furthermore, the finding of *Fictibacillus* in the *An*. *albimanus* gut suggests the importance of conducting studies on its contribution in this mosquito immune response against *Plasmodium*; this because a member of this genus was shown to participate in the signaling of the JAK-STAT pathway [49], which actively intervenes in the *Anopheles aquasalis* immune response against *Plasmodium vivax* [50]. The genera *Paenibacillus*, *Staphylococcus* and *Bacillus* have been linked with male mosquitoes, and differences in microbiota composition have been related with feeding dynamics according to mosquito gender [24]. Given that no blood was detected in the mosquito guts during dissection, it is suggested that the absence of blood favored the presence of Firmicutes in the *An*. *albimanus* female guts. This assumption is also based on the fact that some genera of this phylum are found in the gut of insects which do not perform blood feeding, such as *Drosophila melanogaster* [51] and *Lymantria dispar* [52].

In terms of the influence of mosquito developmental stage on *An*. *albimanus* gut bacterial microbiota, a higher bacterial richness was observed in larvae and there were not dominant morphospecies. Of the 14 genera detected, 11 were present in mosquito larval guts from both localities and of 28 morphospecies isolated, 23 were from larvae. The higher bacterial richness observed in larval stage as compared to the adults for both collection localities is consistent with reports for other *Anopheles* species [13] [22] [24] [25], and it is attributed to the influence of the aquatic environment in which larvae develop [53] [54]. In adults, the loss of the peritrophic membrane during metamorphosis and their restricted feeding result in a lower gut bacterial richness [2] [54]. Similarly, a larger number of OTUs were detected in field or laboratory-reared *An*. *gambiae* larvae with respect to adult mosquitoes under the same conditions [25].

Various studies have reported on a higher abundance of Gram-negative with respect to Gram positive bacteria in the mosquito gut. For example, in the *An*. *stephensi* gut, 12 bacterial genera were detected and from these, five were Gram negative genera [13]. In the present study, *An*. *albimanus* mosquito guts contained similar proportions Gram-positive and Gram-negative bacteria, and morphospecies of the genera *Enterobacter*, *Bacillus* and *Bacillus cereus* Group were more abundant while, *Serratia*, *Acinetobacter*, *Lysinibacillus* and *Staphylococcus* were in lower quantities. In contrast, a study on the gut bacterial microbiota of field and colony *An*. *albimanus* mosquitoes from Mexico only reported the genera *Enterobacter* and *Serratia* and both produced a reduction in *Plasmodium* loads, which was higher with *Serratia* [27].

The present is the first study reporting *Serratia* in Colombian *Anopheles* mosquitoes. Of notice, this bacterium was detected in the *An*. *albimanus* gut with few or no other morphospecies, with only *Acinetobacter* or *Bacillus*. Similarly, a decrease in bacterial richness was reported in *Ae*. *aegypti* when *Serratia* was present [55]. Under laboratory conditions *Serratia* has demonstrated a superior colonizing capacity and permanence in the mosquito guts than other natural bacteria [7]; and of interest, it was found in other mosquito tissues such as the salivary glands [12]. These characteristics make of a bacterium an appropriate candidate for a paratransgenic control. It will be desirable to evaluate which of these properties are present in the *Serratia* isolates from *An*. *albimanus*. Furthermore, *Enterobacter* morphospecies were detected in both developmental stages, but in higher abundance in adults from San Antero. It is argued that the mosquito gut bacteria can be acquired by feeding, trans-stadially or vertically from mother to egg [2]. *Enterobacter* is commonly found in soil and water matrices [56] [57] [58]; therefore, its finding in these specimens suggests an acquisition from the water of the larval habitat and its survival during mosquito metamorphosis. However, an acquisition through feeding by the adult mosquito is also possible because *Enterobacter* is frequently found in plants with nitrogen-fixing activity, and may be acquired by insects during sugar feeding [59] [60] [61]. Similar to the *Serratia* genus, some *Enterobacter* species can reduce the *Plasmodium* parasite in *Anopheles* mosquitoes [9] [10]. Future studies should be directed to characterize the *Enterobacter* and *Serratia* morphospecies, to evaluate their biological functions in the *An*. *albimanus* mosquito gut and potential antiparasitic activity.

Regarding the influence of mosquito geographical origin in the composition of *An*. *albimanus* gut bacterial communities, a higher bacterial richness was detected in mosquitoes and larvae from Buenaventura-Pacific Coast with respect to San Antero-Atlantic Coast. Studies on *Anopheles* gut microbiota have reported differences in microbial composition according to sampling locality, in addition, the variable geography had a stronger influence on bacterial diversity than the variable mosquito species [62] [63].

As such, differences in gut microbiota were documented for *An*. *gambiae* and *An*. *coluzzi*, with sampling location positively influencing their bacterial communities diversity [62]. In the present study, the bacterial genera shared among the *An*. *albimanus* specimens from both sampled localities were *Bacillus*, *Lysinibacillus* and the *Bacillus cereus* Group. These bacteria are commonly found in water, soil and plants, which suggests that they may have been acquired from the environment. Meanwhile, the genera *Aeromonas*, *Kurthia*, *Esherichia-Shigella*, *Klebsiella* and *Micrococcus* were unique to San Antero, while *Serratia*, *Fictibacillus*, *Staphylococcus*, *Paenibacillus* and *Acinetobacter* were only detected in specimens from Buenaventura. Furthermore, genera such as *Enterobacter* and *Serratia* detected in the specimens were also found in *An*. *albimanus* from Mexico [27]; *Acinetobacter*, *Enterobacter* and *Klebsiella* in *An*. *albimanus* from Peru [64], and *Enterobacter* and *Bacillus* in various developmental stages of *An*. *albimanus* specimens from Colombia [53]. According to the recently proposed panmicrobiota theory, referring to the microbiota present in different mosquitoes independent of geography [65], it will be of interest to evaluate if morphospecies of these genera are part of this panmicrobiota.

Regarding bacterial diversity, minor differences in Equitability (0.94 to 0.96) and Dominance (D) (0.08 to 0.1) were detected for the *An*. *albimanus* adults and larvae from both localities and mosquito stages. Studies on mosquito microbiota suggest that conditions in the gut such as pH and oxygen tension modulate a similar bacterial communities diversity [66]. In addition, the Shannon index showed uniformity in morphospecies distribution in *An*. *albimanus* larvae and adults, and in both localities, indicating a homogeneous distribution of morphospecies in the specimens. Nonetheless, *Bacillus cereus* Group morphospecies were frequent in the Buenaventura mosquitoes; they were found in more than 50% of the specimens, while they were rare in larvae. Similar results were reported regarding the gut bacterial microbiota in *Anopheles* mosquitoes from Vietnam, in which *Bacillus* and other genera of the Firmicutes, e.g. *Staphylococcus* were detected in abundances higher than 50% [42]. Meanwhile, in San Antero locality the uniformity in morphospecies distribution was more equitable with a minor increase in *Enterobacter* M1 in adults. Slight variations in bacterial morphospecies distribution have been attributed to particular conditions of specimens, such as diet variations, bacterial or parasites infections [25] [66] and also, to intrinsic conditions such as variations at the genetic level [67].

Bacteria such as *Acinetobacter* and *Aeromonas* found as part of *An*. *albimanus* gut microbiota were previously reported in two other Colombian main malaria vectors, *Anopheles darlingi* and *Anopheles nuneztovari* [22]. If upon evaluation of these bacteria they were found with paratransgenic properties, their presence in various Colombian vector species would suggest a facilitated dispersion among the mosquito communities; they will be more susceptible to incorporate the bacteria after reintroduction. Their capacity to colonize and remain among the bacterial communities independent of the *Anopheles* species and geographical location will guarantee their success in a paratransgenic approach. Furthermore, morphotypes of the genera *Enterobacter*, *Klebsiella*, *Aeromonas*, *Bacillus* and *Serratia* were found with natural resistance to antibiotics commonly used for colonization tests, such as ampicillin. This resistance property facilitates conducting *in vitro* and *in vivo* experimentation and mosquito gut-colonization tests. Obtaining these bacterial isolates naturally present in the *An*. *albimanus* gut is an initial basic step; the challenge for archiving a paratransgenic approach is the detection of antiparasitic molecules to potentiate and use in the natural environment, achieving their dispersal. The available culture collection rises the possibility of evaluating the isolates, e.g. *Enterobacter*, *Klebsiella*, *Aeromonas*, *Bacillus* and *Serratia* in colonized *An*. *albimanus*, to evaluate their potential antiparasitic activity and dispersion ability towards other main Colombia vector species such as *An*. *nuneztovari* and *An*. *darlingi*.

## Conclusions

This study demonstrated that the characterization of morphospecies by culture-dependent methods complemented with 16S rRNA gene sequencing allows a finer resolution of the composition and structure of the microbial communities. Interestingly, some of the bacterial genera detected have been previously described in other *Anopheles* mosquitoes showing potential for *Plasmodium* parasite control; therefore, the availability of a bacterial culture collection will allow performing functional tests to prove their potential for a paratransgenic control.

**Funding sources:** This study was funded by Universidad de Antioquia, project Code No. 2015–7543.

### Availability of data

The datasets generated and analyzed during the current study are available in the GenBank repository, under the accession numbers MK751368-MK751369, MK715480-MK715484, MK704400, MK615614.

## Supporting information

S1 Table Morphospecies taxonomic assignment. Taxonomic classification of bacterial morphospecies and their distribution by locality and stage. X: designates presence in the evaluated group, O: absence, L: Larvae, A: Adults, SAN: San Antero (Atlantic Coast), BUE: Buenaventura (Pacific Coast).
(DOCX)

S1 Fig Dendrogram generated from Jaccard distances using the UPGMA algorithm with 1000 replicas. Distribution of the bacterial genera in larvae and adults, intra-locality. (A) San Antero (Atlantic Coast). (B) Buenaventura (Pacific Coast). Larva and mosquito figures indicate the stage where genera were detected.
(TIF)

S2 Fig Dendrogram generated from Jaccard distances using the UPGMA algorithm with 1000 replicas. Distribution of the bacterial genera by stage. (A) larvae. (B) adult mosquitoes, and according to locality.
(TIF)
