# Supplementary material for: Composition and structure of the culturable gut bacterial communities in Anopheles albimanus from Colombia
Source: PLoS One. 2019 Dec 2;14(12):e0225833. doi: 10.1371/journal.pone.0225833 (PMC6886788; doi:10.1371/journal.pone.0225833)
Supplement: S1 Table — Taxonomic classification of bacterial morphospecies and their distribution by locality and stage. X: designates presence in the evaluated group, O: absence, L: Larvae, A: Adults, SAN: San Antero (Atlantic Coast), BUE: Buenaventura (Pacific Coast). (DOCX) [file pone.0225833.s001.docx]

| **Genera** | **Morphospecies** | **Stages** | | | | **Localities** | |
| --- | --- | --- | --- | --- | --- | --- | --- |
|  |  | **SAN** | | **BUE** | |  |  |
|  |  | **L** | **A** | **L** | **A** | **SAN** | **BUE** |
| *Bacillus cereus* Group | M1 | O | X | X | X | X | X |
|  | M2 | O | X | X | X | X | X |
|  | M3 | O | O | X | X | O | X |
| *Bacillus* | M1 | X | X | X | X | X | X |
|  | M2 | X | X | X | X | X | X |
|  | M3 | O | O | X | X | O | X |
|  | M4 | O | O | X | X | O | X |
| *Fictibacillus* | M1 | O | O | X | O | O | X |
|  | M2 | O | O | X | O | O | X |
| *Staphylococcus* | M1 | O | O | X | X | O | X |
|  | M2 | O | O | X | X | O | X |
|  | M3 | O | O | X | O | O | X |
| *Lysinibacillus* | M1 | X | O | O | O | X | O |
|  | M2 | O | O | X | X | O | X |
| *Kurthia* | M1 | X | O | O | O | X | O |
|  | M2 | X | O | O | O | X | O |
| *Paenibacillus* | M1 | O | O | X | O | O | X |
| *Serratia* | M1 | O | O | O | X | O | X |
|  | M2 | O | O | O | X | O | X |
| *Acinetobacter* | M1 | O | O | O | X | O | X |
| *Enterobacter* | M1 | X | X | O | O | X | O |
|  | M2 | X | X | O | O | X | O |
|  | M3 | X | O | O | O | X | O |
| *Escherichia-Shigella* | M1 | X | O | O | O | X | O |
| *Klebsiella* | M1 | X | O | O | O | X | O |
| *Aeromonas* | M1 | X | O | O | O | X | O |
|  | M2 | X | O | O | O | X | O |
| *Micrococcus* | M1 | X | O | O | O | X | O |
