# Supplementary figures and images for: Composition and structure of the culturable gut bacterial communities in Anopheles albimanus from Colombia
Source: PLoS One. 2019 Dec 2;14(12):e0225833. doi: 10.1371/journal.pone.0225833 (PMC6886788; doi:10.1371/journal.pone.0225833)

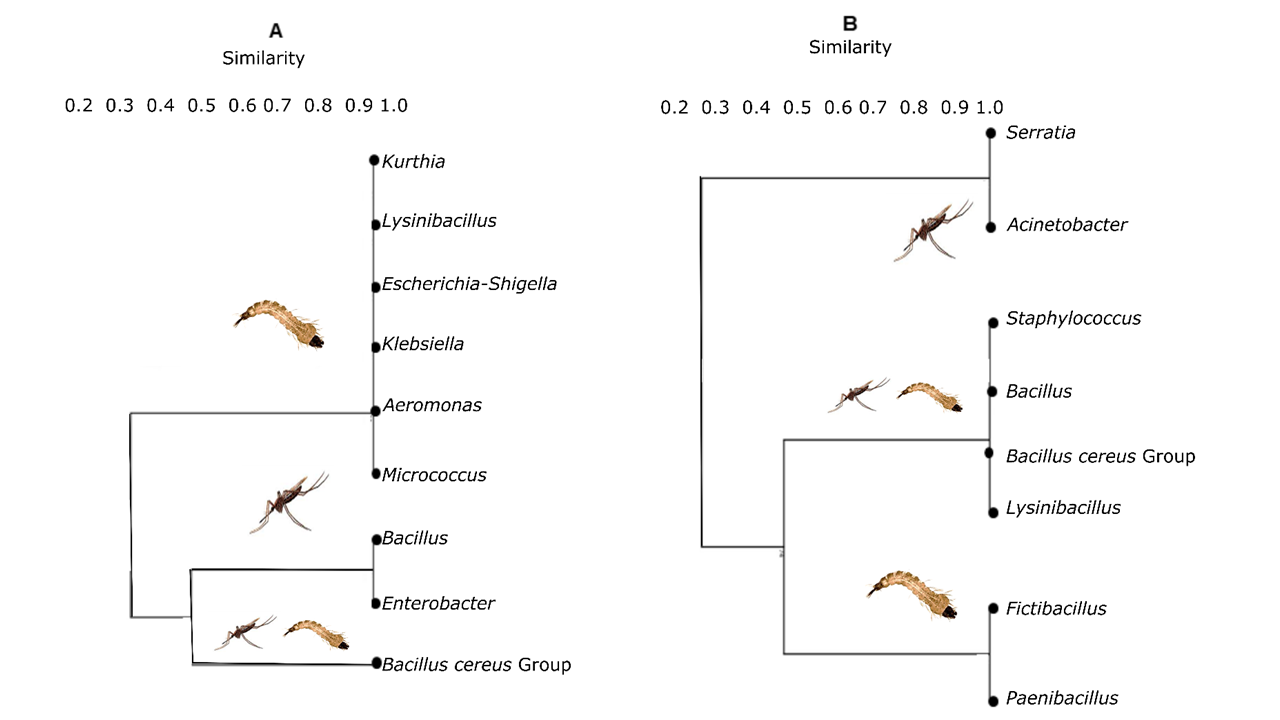

Supplement: S1 Fig — Distribution of the bacterial genera in larvae and adults, intra-locality. (A) San Antero (Atlantic Coast). (B) Buenaventura (Pacific Coast). Larva and mosquito figures indicate the stage where genera were detected. (TIF) [file pone.0225833.s002.tif]

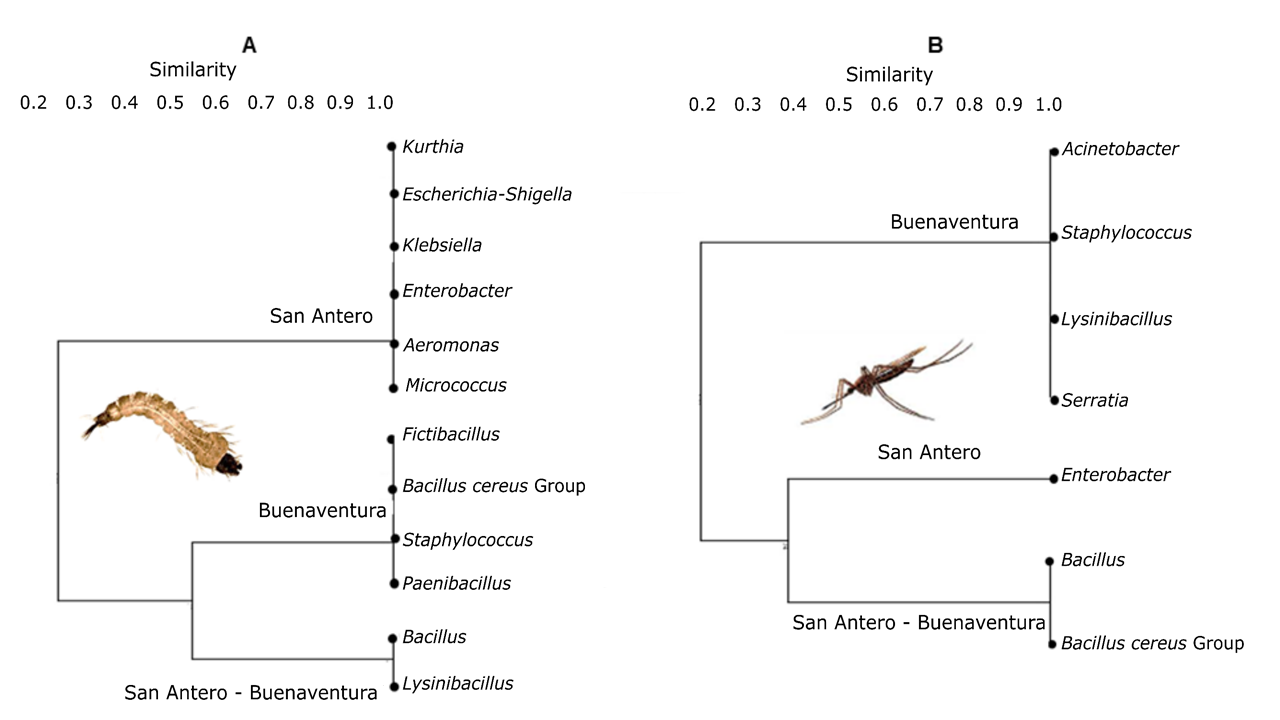

Supplement: S2 Fig — Distribution of the bacterial genera by stage. (A) larvae. (B) adult mosquitoes, and according to locality. (TIF) [file pone.0225833.s003.tif]
